# Prospective Study of Intensity-Modulated Radiation Therapy for Locally Advanced Breast Cancer

**DOI:** 10.3390/cancers12123852

**Published:** 2020-12-20

**Authors:** Benoît Bataille, Bennadji Raoudha, Florence Le Tinier, Laurent Basson, Alexandre Escande, Hélène Langin, Emmanuelle Tresch, Frederik Crop, Franck Darloy, Damien Carlier, Eric Lartigau, David Pasquier

**Affiliations:** 1 Academic Department of Radiation Oncology, Oscar Lambret Comprehensive Cancer Center, 59020 Lille, France; b-bataille@o-lambret.fr (B.B.); b.raoudha@gmail.com (B.R.); f-letinier@o-lambret.fr (F.L.T.); laurentbasson@yahoo.fr (L.B.); a-escande@o-lambret.fr (A.E.); h-langin@o-lambret.fr (H.L.); e-lartigau@o-lambret.fr (E.L.); 2 Methodology and Biostatistics Department, Oscar Lambret Comprehensive Cancer Center, 59020 Lille, France; e-tresch@o-lambret.fr; 3 Medical Physics Department, Oscar Lambret Comprehensive Cancer Center, 59020 Lille, France; f-crop@o-lambret.fr; 4 Department of Radiation Oncology, Leonard De Vinci center, 59187 Dechy, France; fdarloy@clinique-psv.fr (F.D.); dcarlier@clinique-psv.fr (D.C.); 5 CRIStAL UMR CNRS 9189, Lille University, 59000 Lille, France

**Keywords:** breast cancer, IMRT, tomotherapy, simultaneous integrated boost, quality of life

## Abstract

**Simple Summary:**

Tomotherapy is a modern intensity-modulated radiotherapy technique, whose aim is to reduce the high doses delivered to organs at risk. Thus, we conducted a prospective study evaluating the early and medium-term toxicities, the patient’s quality of life, and the aesthetic outcomes (evaluated by both physicians and patients) of tomotherapy for breast cancer. We wanted to show that this treatment is very well tolerated, with low-grade acute toxicities, and has only a moderate impact on patients’ quality of life and aesthetic outcome, in order to support its larger use in this very frequent situation.

**Abstract:**

The objective of this study was to evaluate the acute and medium-term toxicities, the quality of life, and aesthetic results of patients with breast cancer (BC) treated with tomotherapy. This was a prospective study, including patients with BC treated by tomotherapy. Radiation therapy delivered 50 Gy in 25 fractions to the breast or chest wall and to lymph node areas, with a simultaneous integrated boost at a dose of 60 Gy at the tumor bed in cases of breast conservative surgery. We included 288 patients, 168 and 120 treated with breast-conserving surgery and mastectomy respectively. Two hundred sixty patients (90.3%) received lymph node irradiation. Median follow-up was 25 months (6–48). Acute dermatitis was observed in 278 patients (96.5%), mostly grade 1 (59.7%). The aesthetic aspect of the breast at one year was reported as “good” or “excellent” in 84.6% of patients. The patients’ quality of life improved over time, especially those treated with chemotherapy. The two-year overall survival and disease-free survival were 97.8% (95% confidence interval (CI): 94.1–99.2%), and 93.4% (95% CI: 89.2–96.0%) respectively. Tomotherapy for locally advanced BC has acceptable toxicity, supporting its use in this indication; however, longer follow-up is needed to assess long-term outcomes.

## 1. Introduction

Breast cancer (BC) is the leading cause of cancer-related death in women in developed countries. Patients often receive radiation therapy (RT) after breast-conserving surgery (also known as lumpectomy). After mastectomy, it is performed in the case of nodal involvement, large tumors > 5 cm, unfavorable prognostic factors in a T2 stage tumor: grade 3, lymphovascular space involvement, or young age, according to national guidelines. Three-dimensional conformal radiotherapy (3D-CRT) is the most commonly used technique for adjuvant radiation therapy in breast cancer patients. However, mounting evidence suggests that the use of intensity-modulated radiotherapy (IMRT) might be beneficial for BC in the adjuvant setting. Thanks to its dosimetric benefits, it provides improved dose homogeneity and reduction of the high dose delivered to other organs that are not the intended target, such as the lungs or heart [1]. The clinical advantages of IMRT have been highlighted by three independent randomized trials, which showed improvement in early and late toxicities, quality of life, and aesthetic evaluation [2,3,4]. However, these studies suffered from some limitations. First, the control group received conventional radiotherapy, which is no longer the standard treatment. Moreover, in these trials, IMRT was used as a static technique. Finally, in all three trials, patients had localized BC with no nodal involvement, requiring radiation therapy only to the breast and not to the nodal target volume, which is when the dosimetric advantage seems to have a greater effect [5,6].

In parallel, hypofractionated radiotherapy has been applied in BC for many years now. In terms of survival and toxicity, it has shown equivalence to normal-fractionated radiotherapy in early-stage BC [7], and has benefits regarding accessibility and medical costs. The use of IMRT has allowed the application of simultaneous integrated boost (SIB) to the tumor bed, with the delivery of two different doses per fraction in every radiotherapy session [8]. Limited prospective data exist regarding the use of SIB with conventional fractionation regimens for the treatment of BC; however, a low occurrence of high-grade toxicities was reported [9,10,11].

The purpose of this prospective study was to assess the acute tolerance of BC patients treated with tomotherapy. As secondary objectives, medium-term tolerance, aesthetic evaluation, quality of life, and survival were evaluated.

## 2. Patients and Methods

This study was a prospective, bicentric, routine-care study aimed at evaluating the safety of tomotherapy treatment for patients with localized BC previously treated by surgery (clinicalTrials.gov identifier: NCT02281149). The study was approved by the local ethics committee (“Comité de Protection des Personnes Nord Ouest IV”) and conducted in accordance with the Helsinki declaration and good clinical practice guidelines. Informed consent was obtained from all patients prior to enrollment in the study.

### 2.1. Inclusion/Exclusion Criteria

Patients aged 18 years or more with histologically proven BC treated by adjuvant radiation therapy after either conservative or not conservative breast surgery were included in this study.

Patients diagnosed with metastatic disease, who were pregnant or breast-feeding, who had severe or noncontrolled pathology that would compromise participation in the trial, or those unable to undergo medical follow-up due to geographic, medical, or psychological reasons were excluded from this study.

### 2.2. Endpoints

The study’s primary endpoints were acute side effects (occurring within 90 days after the end of radiotherapy). The side effects were evaluated according to the National Cancer Institute Common Terminology Criteria for Adverse Effects (NCI-CTCAE) v4.0.

The study’s secondary endpoints included medium-term and long-term side effects (occurring more than 90 days after the end of radiotherapy) and quality of life, which was evaluated using the QLQ-C30 and QLQ-Br23 questionnaires from the European Organization for Research and Treatment of Cancer (EORTC). The aesthetic score was determined as excellent, good, average, or mediocre, and the aesthetic appearance was scored by patients and physicians independently. Disease-free survival and overall survival were also recorded as a secondary endpoint.

### 2.3. Treatment Procedure

Patients with non-metastatic BC received tomotherapy in the adjuvant setting. The whole treatment procedure has already been detailed in previous studies [12,13,14]. Before treatment, patients underwent a planning computer tomography (Toshiba medical system, Japan), with an axial slice thickness of 2.5 mm. Immobilization was obtained with a MedTec system (CIVCO Medical solutions, Coralville, IA, USA) in a supine position. Radiation therapy consisted of a helical intensity-modulated radiation therapy with 6MV photons. Treatment planning was performed by a TomoTherapy Hi-Art system (Accuray, Sunnyvale, CA, USA) using an inverse planning method. Dose constraints to organs at risk were defined as follows: ipsilateral lung: V15 Gy < 50%, V20 Gy < 35%, V30 Gy < 20%, V35 Gy < 15%; contralateral lung: V10 Gy < 50%, V12 Gy < 35%, V15 Gy < 20%; heart (for left breast cancer): V15 Gy < 20%, V20 Gy < 15%, V25 Gy < 10%; contralateral breast: V5 Gy < 50%, V7 Gy < 35%, V10 Gy < 20%, V20 Gy < 15%. The target volume was defined according to the ASTRO guidelines until 2016, and to the ESTRO guidelines thereafter [15,16]. Planned target volume (PTV) was obtained by adding a 5 mm margin to the clinical target volume (CTV). The prescribed doses were 50 Gy in 25 fractions of 2 Gy to the breast or chest wall and nodal target volume if indicated, and 60 Gy in 25 fractions of 2.4 Gy to the tumor bed in cases of breast-conserving surgery using SIB. The aim was that 95% of the volume received 95% of the prescribed dose. Daily image-guided radiation therapy (IGRT) was performed using daily megavoltage CT scans (MVCT) and/or surface IGRT (Catalyst), which was automatically registered to the CT scan; position was corrected by experienced staff members if necessary. Patients were followed up with each week during radiotherapy, at 1 and 6 months after radiotherapy, and then annually. Quality of life was assessed at admission, at 1 and 6 months after radiotherapy, and then annually.

### 2.4. Statistics

The number of subjects included in this study was based on its primary objective, that is, acute toxicity of grade ≥2 (considered a failure). When searching for prognostic factors, at least 10 failures per prognostic factor should be observed. In this study, we planned to study 9 factors (age, BMI, smoking, diabetes, cosmetic outcome, previous chemotherapy, breast volume, D2% of target volume, dose to the skin). For a multivariate analysis with 9 variables, it was necessary to observe 90 patients with grade ≥2 toxicity. According to the literature, it was estimated that the rate of grade ≥2 toxicity was 30%, which corresponds to a required sample size of 300 patients. The association between toxicity and dosimetric characteristics will be presented in a subsequent paper.

The statistical analyses were performed using Stata v15.0 software (StataCorp. 2017. Stata Statistical Software: Release 15. College Station, TX: StataCorp LLC). Relapse-free survival and overall survival were estimated using the Kaplan–Meier method. For these analyses, the time from admission to the time of relapse or death (regardless of cause) was taken into account. Patients alive without recurrence were censored on the latest follow-up date. Median follow-up was calculated by the reverse Kaplan–Meier method.

The scores from the QLQ-C30 and QLQ-Br23 questionnaires corresponding to quality of life were calculated using the EORTC method at admission and at the different follow-up times. Differences between follow-up scores and those from admission were also calculated and described by their medians, extreme values, means, and standard deviations. The evolution of the score over time was analyzed using a mixed model for repeated measures. Admission mean scores of quality of life were compared using a Student’s *t* test.

## 3. Results

### 3.1. Patients

We included 300 patients in this interim analysis, which ran from November 2014 to January 2018. One patient was secondarily excluded because she received hypofractionated radiation therapy. Eleven patients were secondarily excluded from the analysis because they did not receive any treatment. The analysis included the remaining 288 patients, 168 and 120 treated with breast-conserving surgery and mastectomy respectively. Median age at diagnosis was 55 years (range: 32–82). Median follow-up was 25 months (6–48). Patient and tumor characteristics are detailed in Table 1.

### 3.2. Treatment

An axillary evaluation was performed in every case; 176 patients (61.1%) underwent sentinel lymph node dissection, and 205 patients (71.2%) underwent axillary dissection. Re-intervention was required for 54 patients (18.8%); 21 patients (7.3%) underwent a second breast-conserving surgery, and 18 patients (6.3%) underwent axillary dissection alone.

Two hundred nine patients (72.6%) received chemotherapy: 50 (17.4%) before surgery, 148 (51.4%) in an adjuvant setting, and 11 (3.8%) received both neoadjuvant and adjuvant chemotherapy. Anti-HER2 (trastuzumab) treatment was administered to 57 patients (19.8%). Two hundred twenty-seven patients were treated with adjuvant hormone therapy (80.2%).

Lymph node irradiation was performed in 260 patients (90.3%). The median duration of treatment was 36 days (range: 33–45). Eighty-eight patients (30.6%) had a delineation of target volumes according to the RTOG recommendations and 200 patients (69.4%) according to the ESTRO consensus.

Doses delivered to target volumes and organs at risks are summarized in Table 2 and Table 3.

### 3.3. Toxicity

Acute skin reaction occurred in 278 patients (96.5%), with grade 1 dermatitis in 172 patients (59.7%), grade 2 in 102 patients (35.4%), and grade 3 in 4 patients (1.4%). None of the patients experienced grade 4 or 5 dermatitis. Esophagitis was observed in 138 patients (47.9%); 120 patients (41.7%) with grade 1 and 18 patients (6.3%) with grade 2. Asthenia occurred in 127 patients (44.1%); grade 1 in 114 patients (39.6%), and grade 2 in 13 patients (4.5%). Edema was present in 57 patients (19.8%), including 55 patients (19.1%) with grade 1 and one patient (0.3%) with grade 2.

Regarding medium-term side effects, 152 patients (53.1%) experienced medium-term skin toxicity, including 147 (51.4%) grade 1, two (0.6%) grade 2, and two (0.6%) grade 3 (one radiodermatitis at 6 months and one ulceration-necrosis at 12 months). 69 patients (41.1%) had breast edema, including 63 (37.5%) grade 1, and 5 (3.0%) grade 2. Esophageal toxicity was present in only six patients (2.1%), all with grade 1.

Breast fibrosis was observed in 68 of 168 patients (40.5%) treated with partial mastectomy, including 59 cases (35.3%) with grade 1 and 9 cases (5.4%) with grade 2. None of the patients developed breast fibrosis higher than grade 2.

Finally, among the 168 patients treated with breast-conserving surgery, tumor bed fibrosis was present in 66 patients (39.5%), including 56 cases (33.5%) with grade 1 and 10 cases (6.0%) with grade 2. None of the patients developed lumpectomy bed fibrosis of grade 3 or higher.

In the 120 patients treated with mastectomy, parietal fibrosis was present in 51 patients (42.9%), including 42 cases (35.3%) with grade 1 and 9 cases (5.4%) with grade 2.

Acute and medium-term toxicities are presented in Table 4.

### 3.4. Aesthetic Assessment

The aesthetic assessment was performed only after breast-conserving surgery and was generally consistent between patients and physicians. One year after irradiation, 135 patient aesthetic assessment scores were recorded by physicians and 136 by patients. The patient’s appearance was rated “poor” by one physician (0.7%) and 3 patients (2.2%); “average” by 17 physicians (12.6%) and 18 patients (13.2%); “good” by 64 physicians (47.4%) and 62 patients (45.6%); and “excellent” by 53 physicians (39.3%) and 53 patients (39%). In 88 out of 131 judgments, the assessments by the physician and the patient were identical, and the judgment of aesthetic appearance did not differ significantly (*p* = 0.54; exact symmetry test).

### 3.5. Quality of Life

**Figure 1:**
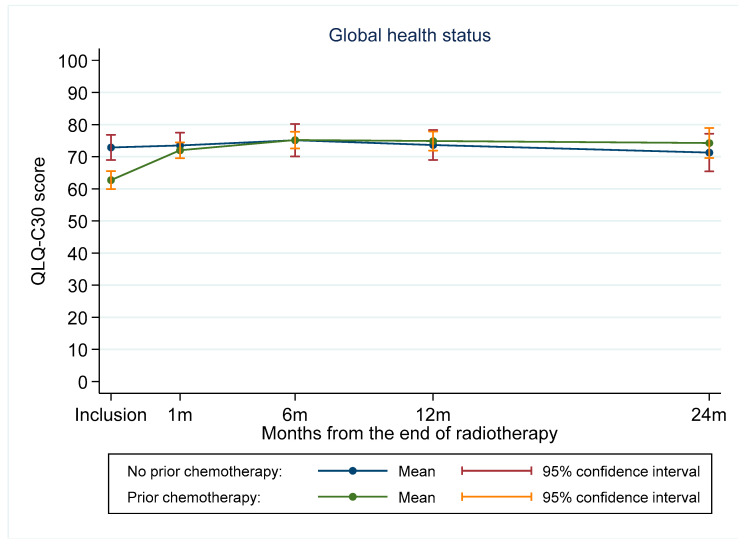
Evolution of quality of life global health score over time according to chemotherapy status (breast-conserving surgery).

In the whole population, scores reflecting overall health and most functional scales increased over time, indicating a better overall quality of life. Chemotherapy had a profound impact on admission scores and their progression over the course of the treatment. The mean inclusion scores were significantly lower in patients who received chemotherapy compared with patients who did not receive it (61.6 vs. 73.0, *p* < 0.001). In patients who did not receive chemotherapy, the overall health status did not change significantly over time (*p* = 0.49), whereas there was an improvement in patients who received chemotherapy (*p* < 0.001). In patients who received chemotherapy, the overall health status increased at one month following the end of radiotherapy, leading to an overlap with the curve from the patients treated by radiotherapy only (Figure 1). For some quality-of-life indicators, there was an initial deterioration in the mean score at one month and six months, possibly related to the radiation-induced acute reactions, followed by an improvement thereafter.

### 3.6. Survival

**Figure 2:**
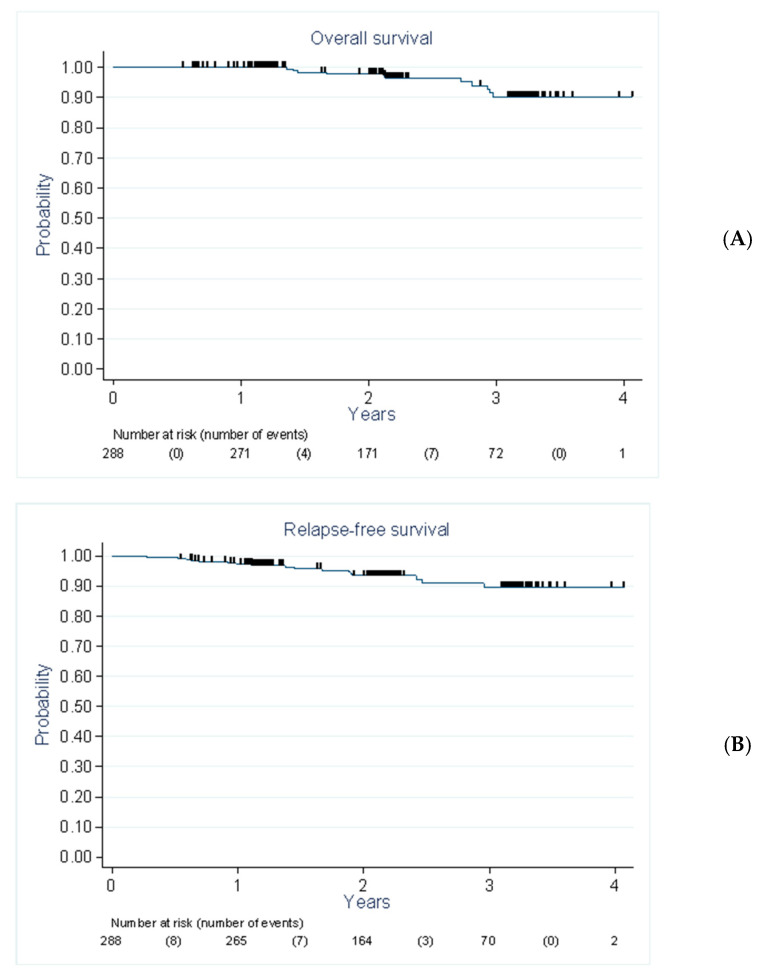
Overall survival (**A**) and disease-free survival (**B**) according to the Kaplan–Meier method (breast-conserving surgery).

At the time of analysis, 11 patients (3.8%) had died, including seven deaths related to BC progression. The overall survival rate at two years was 97.8% (95% CI: 94.1–99.2%) (Figure 2).

Seventeen cases of recurrence were reported: 13 cases of metastatic recurrence only, three cases of local recurrence (all associated with regional and metastatic recurrence), and one case of regional recurrence associated with metastatic recurrence. The two-year disease-free survival rate was 93.4% (95% CI: 89.2–96.0%) (Figure 2).

## 4. Discussion

In this study, we performed a prospective evaluation of toxicities and quality of life in 288 patients treated with tomotherapy for locally advanced BC, using SIB in patients treated with conservative surgery. To our knowledge, our study is one of the few providing extensive prospective data in this setting.

The toxicity results described here are comparable with those in the literature for IMRT treatments in adjuvant BC radiotherapy. Previous studies reported skin toxicity in the majority of cases, with most of them being grade 1 reactions (50–84%); no high-grade (≥3) toxicity has been reported [17,18,19,20,21]. In line with these findings, most of the patients in our study experienced a skin reaction (278/288; 96.5%), which was mostly moderate. However, it is not straight-forward to directly compare our findings to those from previous studies, since these studies were mostly retrospective and used different design and IMRT techniques. IMRT appears to improve acute skin toxicity compared to 3D-CRT, as shown in the study by Freedman et al., which involved 73 patients treated with IMRT matched to 60 patients treated with 3D-CRT. The study showed a significantly higher prevalence of grade 2 moist desquamation in the 3D-CRT group compared to the IMRT group (38% vs. 21%, *p* = 0.001) [22]. Some reports exist regarding esophagitis, yet the number of these reports is very limited. For example, in a retrospective helical IMRT study, Aoulad et al. reported 19.9% of grade 1 and 2 acute esophagitis [21].

IMRT use in BC has been shown to reduce the rate of high-grade fibrosis by improving the homogeneity of the delivered dose in the breast [23]. Thus, the Royal Marsden randomized trial found a statistically significant reduction in breast induration for patients treated with IMRT compared to those treated with two-dimensional radiotherapy (2D-RT) (for the tumor bed: 61% vs. 37%, *p* < 0.001) [2]. However, the two other randomized trials did not find any difference between the two groups for breast fibrosis. In our study, breast, chest wall, or tumor bed fibrosis represented approximatively 40% of patients, without any patients with grade ≥3. The prospective and systematic nature of data collection allowed for a detection of all cases with grade 1 fibrosis, which correspond to a slight induration of the breast, without any functional or aesthetic impact. Moreover, our cohort was composed of a significant proportion of patients with high risk factors for developing breast fibrosis, including weight (33.2% of the patients were overweight), smoking (27.4%), previous chemotherapy (72.6%), irradiation of lymph node areas (78.3%), and boost on the tumor bed (100% of conservative surgical treatments) [24,25]. In the short term, the induration and fibrosis were more related to surgery than to radiotherapy. The assessment of short-term fibrosis probably increased the estimated incidence.

In addition, our prospective study was among the first to employ SIB on the tumor bed. Very few prospective studies have tried to assess this technique. In the study by Bantema-Joppe et al., prospective evaluation of an integrated 3D-CRT boost at three years indicated a fibrosis rate of 8.5% in the tumor bed and 49.4% in the breast as a whole [26]. These toxicity rates are significantly higher than what our study has shown, suggesting a potential beneficial effect of IMRT over 3D-CRT. Comparison between SIB and sequential boost showed a reduction in early grade 2 skin reaction in patients who had received IMRT-SIB compared to those who underwent sequential boost with conventional radiotherapy (4.5% vs. 18.3%, *p* = 0.048) [27].

Quality of life is an important concern in patients with BC. Thus, “patient-reported outcomes” (PROS) are usually recorded to get an insight into patients’ feelings about their illness and treatments. In our study, quality of life scores showed a global trend of improving over time. This was largely due to the high proportion of patients treated with chemotherapy (72.6%). Indeed, in the absence of chemotherapy, the indexes corresponding to the overall health status, and most of the symptomatic scale indexes were not significantly altered by postoperative radiotherapy. However, patients who received chemotherapy had a poorer inclusion score than the population without chemotherapy, and the variation in this score was different during follow-up, showing a greater improvement. The impact of chemotherapy is well known and reported in numerous studies, regardless of the radiation therapy technique used [28,29]. In addition, some authors have reported the association between factors related to the patient characteristics and previous treatments (young age, BMI, type of surgery, postoperative complications, post-surgical cosmetic appearance, and axillary cleaning) and quality of life [28,30]. In a prospective cohort of 175 patients treated with 3D-CRT for good prognostic BC (only 27% of whom received chemotherapy), the authors described a significant decrease in the quality of life (assessed by QLQ-C30 and QLQ-BR23 questionnaires) in the period immediately following irradiation, which improved thereafter [31].

Interestingly, we found that patients treated with radiotherapy only (no chemotherapy) did not experience any changes in overall health status, highlighting the high safety of tomotherapy. However, in the three randomized trials that compared IMRT to 2D-RT, there was no statistically significant difference in the quality of life between the two groups [2,3,4]. This may be explained by the fact that only certain indexes of the EORTC questionnaires correspond to specific toxicities of irradiation. The rest are mainly indicative of the overall quality of life and characteristics that can be linked to other treatments (chemotherapy, hormone therapy, or surgery). Thus, in order to improve the quality of life of BC patients, a multidisciplinary and comprehensive approach should be adopted, since the impact of different treatments seems to be inseparable.

Dosimetric parameters are very important to anticipate potential short-term or long-term toxicities. As expected, in our study, tomotherapy provided few high doses to surrounding organs at risk, but was also responsible for a higher volume of organs at risk receiving low doses. For example, mean radiation therapy doses delivered to the heart during treatment of a left breast cancer with this technique is 7.8 Gy (SD = 3), and the volume of the heart receiving 40 Gy is only 0.5% (SD = 0.6). As patients in this study mostly received lymph node irradiation (84.5%), the total treatment volume is important, and leads to those results. As an example, a dosimetric study from *Caudrelier* et al. compared tomotherapy to 3D-CRT for breast radiotherapy with lymph node involvement, and showed a diminution of high doses delivered to the lung by 22% for the volume receiving 20 Gy, and 23% for the volume receiving 30 Gy [6].

This study has several limitations. First, our population included patients treated by either breast-conserving surgery or mastectomy, with or without chemotherapy, concomitant boost, or lymph node irradiation. However, the majority of patients had a locally advanced disease (invaded axillary area in 78.3% of cases, chemotherapy in 72.6% of cases, lymph node irradiation performed in 90.3% of cases). This can lead to a higher rate of acute and medium-term complications because of the larger volumes treated in the event of lymph node involvement. However, despite this being a drawback, it makes the cohort more comparable to the real-life population, which is often different from that of clinical studies in which patients are selected according to specific criteria.

Additionally, despite better target coverage and reduction of high doses in adjacent organs at risk, IMRT provides the delivery of lower doses in adjacent structures such as the contralateral lung and breast. This could lead to an increased risk of developing secondary cancer [32]. Tomotherapy could lead to an increase in low doses delivered to the ipsilateral lung and heart whereas a two-field technique avoids it by using a block beam. However, none of the models estimating the risk of a second cancer have been validated to date and the role of low doses in adults has not yet been established. *Grantzau* et al. assessed the effects of the delivered radiation dose to the lung and the risk of second primary lung cancer. For patients diagnosed with second primary lung cancer, the rate of lung cancer increased linearly with the dose but no increased risk was found for doses below 15–24 Gy [33]. Finally, the relatively short follow-up time (median: 2.1 years) did not provide information on late toxicities, especially cardiac and lung chronic side effects, which are expected to be very late events. Some prospective and retrospective studies have been published with a follow-up similar to that of this study [17,21]. One of the secondary objectives of our study was to assess long term toxicity. Follow-up is ongoing, and further results are expected for late side effects. Further data on late toxicities, recurrence-free survival, and second cancers will be available in the coming years.

## 5. Conclusions

Adjuvant BC radiation therapy by tomotherapy provides low early and medium-term toxicities with encouraging aesthetic and quality of life results; however, longer follow-up is required to sufficiently assess late outcomes.

## Figures and Tables

**Table 1:** Patient characteristics.

Characteristics	Number (%)	Median (Min–Max)
Age		55 (32–82)
Smoking	79 (27.4%)	
Cardiac disease	94 (23.8%)	
Diabetes	43 (14.9%)	
Dyslipidemia	91 (28.3%)	
Respiratory disease	29 (10.1%)	
BMI (Kg/m^2^)		26.5 (16.5–48.8)
Normal	111 (38.8%)	
Overweight (≥25)	95 (33.2%)	
Obese (≥30)	80 (28%)	
WHO score		
0	222 (79%)	
1	58 (20.6%)	
2	1 (0.4%)	
Breast size		
Small (85A-B, 90A)	31 (11.6%)	
Medium (85C, 90B-C, 95A-B)	76 (28.4%)	
Big (>85C, >90C, >95B)	161 (60.1%)	
Breast side		
Left	140 (48.6%)	
Right	132 (45.8%)	
Bilateral	16 (5.6%)	
Size (mm)		
Clinical		30 (0–300)
Imaging		24 (3–80)
Tumor stage		
pT0	23 (8%)	
pT1	122 (42.4%)	
pT2	110 (38.2%)	
pT3	30 (10.4%)	
pT4	3 (1%)	
Nodal status		
pN0	60 (20.8%)	
pN1-3	215 (74.7%)	
pNx	13 (4.5%)	
Histology		
Invasive ductal carcinoma	217	75.4%
Invasive lobular carcinoma	33	11.4%
Other	38	13.2%
In situ component (N = 281)	116	41.3%
SBR (N = 264)		
SBR I	64	24.2%
SBR II	143	54.2%
SBR III	57	21.6%
Lymphovascular space involvement (N = 247)	77	31.2%
ER+ (N = 286)	243	85.0%
PR+ (N = 286)	205	71.7%
HER2+++ (N = 273)	42	15.4%
Triple negative (N = 288)	28	9.7%

Abbreviations: BMI = body mass index; mm: millimeter; T: tumor status according to TNM stage; N: nodal status according to TNM stage; ER: estrogen receptor; PR: progesterone receptor; SBR: Scarff–Bloom–Richardson grade.

**Table 2:** Doses delivered to target volumes.

Heading	D2%: Median (Min–Max)	D50%	D98%
PTV breast (including boost)	61.3 (50.6–63.4)	50 (44.7–51.4)	46 (40.2–49.7)
PTV internal mammary chain	52.3 (46.8–59.6)	49.8 (44.4–51.1)	46.5 (10.6–49)
PTV supraclavicular (area 1 optional, area 2)	52 (46.5–54.3)	49.8 (44.6–51.3)	46.2 (41.9–49.4)
PTV infraclavicular (areas 3–5)	52.0 (46.5–55.2)	49.8 (44.8–51)	46.3 (39–49.6)

PTV: planning target volume; Dx%: dose delivered in x% of the volume; areas 1–5 according to ESTRO guidelines.

**Table 3:** Doses delivered to organs at risk.

Organ at Risk	Dosimetric Parameters		
Spinal cord	D_mean_: 4.7 (1.8)	D2%: 14.8 (5.5)	
Esophagus	D_mean_: 11.4 (4.3)	V30: 9.5 (8.9)	V45: 1.2 (2.5)
Heart (left breast)	D_mean_: 7.8 (3)	V15: 11.3 (7)	V40: 0.5 (0.6)
Ipsilateral lung	D_mean_: 13.5 (2.7)	V20: 24.1 (7)	V30: 14 (4.8)
Contralateral lung	D_mean_: 4.8 (1.6)	V15: 1.9 (2.3)	V20: 0.5 (0.9)
Contralateral breast	D_mean_: 3.8 (1.4)		

D_mean_: mean dose delivered to the organ at risk (Gy) (standard deviation); Vx: Volume receiving x dose (in Gray).

**Table 4:** Acute and medium-term toxicities.

Toxicity	Acute: Number (%)	Medium-Term: Number (%)
Skin reaction	278 (96.5%)	152 (53.1%)
Grade 1	172 (59.7%)	147 (51.0%)
Grade 2	102 (35.4%)	2 (0.6%)
Grade 3	4 (1.4%)	2 (0.6%)
Unknown grade	0	1 (0.3%)
Esophagitis	138 (47.9%)	6 (2.1%)
Grade 1	120 (41.7%)	6 (2.1%)
Grade 2	18 (6.3%)	0
Breast fibrosis (N = 168)	N/A	68 (40.7%)
Grade 1	N/A	59 (35.3%)
Grade 2	N/A	9 (5.4%)
Chest wall fibrosis (N = 120)	N/A	51 (42.9%)
Grade 1	N/A	42 (35.3%)
Grade 2	N/A	9 (7.6%)
Tumor bed fibrosis (N = 168)	31 (18.8%)	66 (39.5%)
Grade 1	29 (17.6%)	56 (33.5%)
Grade 2	1 (0.6%)	10 (6.0%)
Unknown grade	1 (0.6%)	0

N: number; N/A: not applicable.

## Abbreviations

Breast cancer (BC); Radiation therapy (RT); Three-dimensional conformal radiotherapy (3D-CRT); Two-dimensional radiotherapy (2D-RT); Intensity-modulated radiotherapy (IMRT); Simultaneous integrated boost (SIB); European Organization for Research and Treatment of Cancer (EORTC); Planned target volume (PTV); Clinical target volume (CTV); Standard deviation (SD); Patient-reported outcomes (PROS); Gray (Gy).
